# Morphological and Biochemical Abnormalities of Gracilis Muscle from Children with Cerebral Palsy

**DOI:** 10.3390/jfmk11010090

**Published:** 2026-02-22

**Authors:** Vadim Evreinov, Maksim Stogov, Elena Kireeva, Galina Filimonova, Tatyana Zhirova, Margarita Alisa Popkova, Dmitry Popkov

**Affiliations:** 1 National Ilizarov Medical Research Centre for Traumatology and Ortopaedics, Kurgan 640014, Russia; evreinov2020@mail.ru (V.E.); stogo_off@list.ru (M.S.);; 2 Ural Chaklin Institute of Traumatology and Orthopedics, Ekaterinburg 620000, Russia; 3 Biological Chemistry, Johannes Kepler University of Linz, 4040 Linz, Austria

**Keywords:** children, cerebral palsy, gracilis muscle, biochemical and pathomorphological alterations

## Abstract

Background: Developing an evidence base for physiotherapy programs for patients with Cerebral Palsy (CP) requires an understanding of the microscopic and metabolic processes in striated muscle. The gracilis muscle represents a logical object of study due to the significant morphological changes in individuals with cerebral palsy. This research aims to study morphological and biochemical alterations in the gracilis muscle depending on the severity of motor impairments in CP patients. Methods: The cross-sectional study included 24 patients stratified by the severity of motor impairment. Intraoperative gracilis muscle samples were obtained during tenomyotomies. Nutritional status of patients, morphometric, and biochemical parameters were evaluated. Results: Initial body mass and Quetelet index (*p* = 0.02) were lower in GMFCS V patients (*p* = 0.01) compared to GMFCS IV and GMFCS II-III. Muscle tissue predominated in histological samples of GMFCS II-III and GMFCS V patients (*p* = 0.79), while connective tissue content was higher in the GMFCS IV group (*p* = 0.03). Strong, fast-twitch, anaerobic fibers (*p* = 0.761) with reduced creatine phosphokinase activity (*p* = 0.012) were more frequently observed in the intraoperative samples of GMFCS V patients. Low creatine phosphokinase activity was revealed in children in the GMFCS V group (*p* = 0.012). Conclusions: The structural and metabolic abnormalities observed in gracilis muscle of patients with spastic cerebral palsy indicates profound functional muscular dysfunction, representing one of the factors limiting children’s motor ability. The morphological and biochemical alterations in the striated muscle of CP children correlate with severity of motor dysfunction conditioned by the primary upper motor neuron disorders. Less significant changes in muscles in ambulatory children reflect favorable basis for physical therapy.

## 1. Introduction

Cerebral palsy (CP) is a complex neurological disorder arising from heterogeneous etiological factors, severe comorbid pathology, and versatile clinical manifestations of motor dysfunction and impaired postural control [1]. In 80% of cases, cerebral palsy develops as a result of damage to Betz cells in the cerebral cortex, leading to spasticity, hyperreflexia, and secondary disorders of the musculoskeletal system [2,3]. Secondary alterations in muscle structure and function have long been attributed to encephalopathy, whereas the pathomorphological and biochemical processes in contractile tissues have remained underestimated or controversial [4,5]. Recent studies report decreased satellite cell quantity, disordered sarcomeregenesis, and increased connective and adipose tissue in the endomysium, resulting in the transformation of muscle microstructure [6,7]. Recent studies revealed disrupted ribosome and mitochondrial biogenesis in myosymplasts, an abrupt reduction in the protein-synthetic function of cells, deficiencies in oxidative phosphorylation enzymes, insufficient production of high-energy adenosine triphosphate (ATP) molecules, and a shift in the metabolic profile of muscle fibers toward a predominance of fast myosin isoforms in muscles of CP children [8,9]. These abnormalities limit the physical activity of CP patients by reducing the capacity for mechanical load and exacerbating striated muscle atrophy, thereby promoting secondary sarcopenia [10]. With age, the muscles of the affected limbs decrease in volume, shorten, and weaken, which reduces exercise tolerance and limits the range of motion in the joints [11]. Consequently, every third patient with cerebral palsy develops hip joint contractures (36–64%), pathologically caused by shortening of the adductor muscles, including the gracilis muscle [10,11]. Orthopedic treatments of this condition aim to increase the passive range of motion in the joint and preserve skeletal musculature contractility, implemented via muscle-lengthening procedures, including the gracilis muscle [12,13]. Performing a tenomyotomy on the gracilis muscle allows a lengthening of this muscle enabling the acquisition of intraoperative tissue samples for morphological and biochemical study [12]. Consequently, several authors use this anatomical structure as a model for detecting cellular and extracellular abnormalities in striated muscle and subsequently extrapolate the findings to the entire muscular system of CP patients [14,15,16,17,18].

Physical therapy is widely used in the complex treatment of children with spastic cerebral palsy to improve muscle structure and function, joint range of motion, and reduce contractures [2,14,15]. However, there is no consensus regarding the effectiveness of these therapeutic approaches [16,17]. The majority of studies on contractile tissue pathology in CP have analyzed patients with mild to moderate motor disorders, excluding children with severe functional limitations [11,14]. Therefore, developing an evidence base for physiotherapy programs requires an understanding of morphological and metabolic processes in striated muscle [14,15,18]. The gracilis muscle is justifiably chosen as the object of study, as it represents a muscle largely involved in CP secondary disorders with significant morphological changes [19,20,21]. However, there is an important gap in understanding the correlation between biochemical changes in energy metabolism and morphological impairments depending on GMFCS levels.

Thus, understanding the structural and biochemical abnormalities in muscle tissue of CP patients will provide an evidence base for tailoring physical activity programs and predicting the long-term effects of comprehensive rehabilitation.

This research aims to study morphological and biochemical alterations in the gracilis muscle depending on the severity of motor impairments in CP patients.

## 2. Materials and Methods

The cross-sectional study included consecutive 24 patients with cerebral palsy (7 girls and 17 boys), stratified by the severity of motor impairments and the presence of hip adduction contractures. Gracilis muscle tenomyotomy was performed as part of multilevel orthopedic surgery. The study was approved by the institutional ethics committee (Protocol No. 2 (70) dated 21 October 2021) and was conducted in accordance with the ethical standards outlined in the Declaration of Helsinki. Informed consent was obtained from all participants or their legal guardians for anonymous publication of research findings. This study was conducted at the National Ilizarov Medical Research Center of Traumatology and Orthopedics from October 2023 to December 2024.

Inclusion criteria:

- Children with moderate to severe forms of CP (Gross Motor Function Classification System (GMFCS) levels II, III, IV, V) [22];
- Participants had no prior history of multilevel orthopedic surgery and had not received botulinum toxin injections within 1 year prior to intervention;
- Adductor contractures of hip joints;
- Gracilis muscle tenomyotomy as a part of multilevel orthopaedic surgery.

Exclusion criteria: Patients with prior surgical interventions on the adductor muscles.

According to the GMFCS, all patients were divided into three groups of eight people each. Children with severe motor impairments, unable to maintain body position or ambulate in wheelchairs without assistance, were classified as the GMFCS level V group. Patients requiring mobility devices and using wheelchairs for transportation were assigned to the GMFCS level IV group, while patients capable of independent ambulation with minor limitations or requiring additional devices were categorized as the GMFCS levels II-III group.

The mean age (M (SD)) was 9 (3) years in GMFCS V, 12 (3) years in GMFCS IV, and 9 (4) years in GMFCS II-III (*p* = 0.16).

Preoperative nutritional status was assessed using the Life Expectancy Project centile charts for children with cerebral palsy based on sex, GMFCS motor function level, height and weight measurements, and Quetelet’s body mass index (BMI) [23]. BMI was calculated using the formula BMI = weight [kg]/height [m^2^], with height determined through limb segmental measurement. The distribution of values obtained from the Life Expectancy Project scales was expressed as standard deviation scores (Z-scores) following percentile conversion [19]. The degree of motor impairments affecting feeding was evaluated using the EDACS (Eating and Drinking Ability Classification System) scale [24].

Muscle specimens were collected intraoperatively following gracilis tenomyotomy. A 0.5 × 0.5 cm muscle specimen was mounted on rigid cardboard while maintaining its natural tension and immersed in 10% neutral buffered formalin. After 2–3 days of fixation and rinsing in running water, the samples underwent histological sectioning, ethanol dehydration, infiltration, and paraffin block embedding. Tissue sections (5–8 μm thickness) were prepared using a Bromm-2218 microtome (LKB, Kiruna, Sweden) and stained with hematoxylin–eosin, Masson’s trichrome, and PTAH methods. Digital images of histological specimens were obtained using the Pannoramic MIDI II BF hardware and software system (3DHISTECH Ltd., Budapest, Hungary) using whole-slide imaging technology in extended focus mode, which captures multiple focal planes and combines them into a single image. Scanning was performed using a 40× objective lens. Descriptive morphological examination of digitalized microslides (morphometry of muscle fibers, sarcomere length, percentage composition of muscle tissue components in the section) was performed using PANNORAMIC CaseViewer software, version 2.4 (3DHISTECH Ltd., Budapest, Hungary). Sarcomeres were measured on longitudinal muscle sections stained with the PTAH method at high magnification. For each muscle sample, the Z-line distances were measured 350–500 times across 15–20 fields of view, and the mean sarcomere length was subsequently calculated.

Another fragment of the intraoperative gracilis muscle sample was processed for biochemical analysis. The tissue underwent erythrocyte removal by washing and homogenized in a chilled 0.03M KCl solution, followed by centrifugation at 14.000× *g* for 15 min using a Beckman Coulter ultracentrifuge (USA). The resulting supernatant (sarcoplasmic extract) was analyzed for total proteolytic activity, lactate dehydrogenase (LDH) activity, creatine phosphokinase (CPK) activity, glucose, and lactate concentrations. The pellet was further processed for myosin extraction using 0.6M KCl solution, with subsequent recentrifugation at 6.000× *g* for 10 min, followed by protein quantification via the Lowry method. LDH and CPK activities, as well as lactate and glucose concentrations, were measured using Vital Diagnostic reagent kits (Russia) on a Hitachi 902 biochemical analyzer (Hoffmann-La Roche, Italy). Total proteolytic activity was assessed by monitoring hemoglobin proteolysis reaction intensity, expressed as the rate of amino acid release per unit time. Enzyme activities were calculated per gram of sarcoplasmic protein, while substrate concentrations were expressed per wet tissue mass.

Evaluation parameters:

- Nutritional status;
- Morphometric characteristics of gracilis muscle (muscle fiber diameter, proportions of muscular, connective, and adipose tissues, vascular density, intramuscular nerve fascicles, sarcomere length in myofibrils, and fast-to-slow fiber ratio);
- Biochemical characteristics of gracilis muscle (total proteolytic activity, LDH/CPK activities, glucose/lactate concentrations, and myosin content).

Data analysis was performed using Stat Plus version 7 software (AnalystSoft Inc., Brandon, FL, USA) and IBM SPSS Statistics 27.0.1 (SPSS Inc., Chicago, IL, USA). The sample size required to ensure sufficient statistical power was determined using a sample size calculator (α = 0.05; power = 80%; www.clincalc.com). For numerical variables meeting Gaussian distribution criteria (Kolmogorov–Smirnov/Lilliefors tests), quantitative data were expressed as mean ± standard deviation (M (SD)). For non-normally distributed variables, the median (Me) with first and third quartiles (Q1 & Q3) were calculated. Group comparisons of numerical variables were performed using either one-way analysis of variance (ANOVA) or the nonparametric Kruskal–Wallis H-test. When statistically significant differences were identified, subsequent multiple comparisons between groups were carried out using the Bonferroni correction. For proportion analyses, Pearson’s chi-square test was applied. A *p*-value < 0.05 was considered statistically significant. A posteriori power analysis (effect size) was conducted by calculating the effect size using the Kruskal–Wallis test with the eta squared (η^2^ = (H − k + 1)/(n − k). Eta squared values were interpreted as follows: 0.01 to <0.06 (small effect), 0.06 to <0.14 (moderate effect), and ≥0.14 (large effect) (https://search.r-project.org/CRAN/refmans/rstatix/html/kruskal_effsize.html, accessed on 4 January 2026).

## 3. Results

All patients (100%) in the GMFCS V group exhibited spastic tetraplegia. The GMFCS IV group demonstrated an equal distribution of spastic diplegia (50%) and tetraplegia (50%). In contrast, the GMFCS II-III group showed spastic hemiplegia in 3/8 children (37.5%) and spastic diplegia in 5/8 (62.5%) (*p* < 0.001).

No statistically significant differences in EDACS scores were observed between the study groups (*p* = 0.33). The most severe motor impairments (EDACS III) were identified in 2/8 children from the GMFCS V group who required modified food consistency (blended or puréed diets, use of feeding cups, etc.), and in 1/8 children from the GMFCS IV group. No patients in the GMFCS II-III group exhibited comparable difficulties. Notably, patients with the most restricted mobility (requiring parent or guardian assistance) demonstrated poorer nutrient absorption, which influenced their weight parameters. The GMFCS V group demonstrated significantly lower baseline body weight compared to GMFCS IV and GMFCS II-III (pairwise comparisons: GMFCS II-III and GMFCS V (*p* = 0.04), GMFCS IV and GMFCS V (*p* = 0.004), GMFCS II-III and GMFCS IV (*p* = 0.36)). Similarly, Quetelet index values were lower in GMFCS V patients (GMFCS II-III and GMFCS V, (*p* = 0.02), GMFCS IV and GMFCS V (*p* = 0.006), GMFCS II-III and GMFCS IV (*p* = 0.62)). Botulinum toxin injections were administered to 12 patients (3 patients classified as level II-III GMFCS and 8 patients of level IV GMFCS, 1 patient of level V GMFCS) at other medical institutions, at least 14 months earlier, but the injection sites, type of drug used, and doses could not be determined from the medical records. Prior to multilevel surgeries, all patients followed individually tailored physical therapy.

Patient characteristics across groups are presented in Table 1.

**Figure 1:**
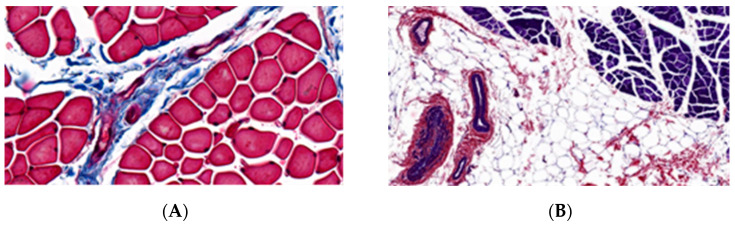

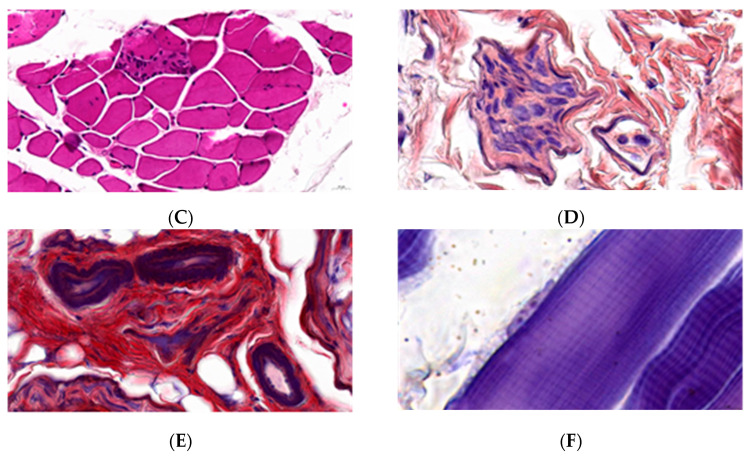
Paraffin-embedded gracilis muscle sections in spastic cerebral palsy: (**A**) (GMFCS II-III group motor impairments)—left part shows polygonal muscle fiber profiles, right part shows size variability of rounded myocytes; (**B**) (GMFCS V group motor impairments)—myosymplasts with size variation, extensive adipocyte deposits, and obliterated vessels; (**C**) muscle fiber myophagocytosis; (**D**) intramuscular nerve fascicle axonopathy; (**E**) sclerosis of perimysial blood vessels; (**F**) myofibril with distinct sarcomeric patterning. Staining: (**A**)—Masson’s trichrome; (**B**,**D**,**E**,**F**)—P.T.A.H.; (**C**)—hematoxylin-eosin. Magnification: (**A**)—720×; (**B**)—130×; (**C**)—500×; (**D**)—1150×; (**E**)—600×; (**F**)—3000×.

Histological findings were consistent with motor impairment level (Figure 1A,B). In cases with severe dystrophic alterations, muscle cells lost their polygonal fiber contours, assuming rounded contours with marked size variation. Nuclei frequently displayed central positioning within the sarcoplasm, with evidence of myophagocytosis of cellular debris (Figure 1C). No statistically significant differences in muscle fiber diameters were observed between the groups (*p* = 0.67), although children with GMFCS V motor impairments frequently demonstrated larger values during morphometric analysis. The gracilis muscle specimens exhibited adipose degeneration, endomysial and perimysial fibrosis, intramuscular nerve fascicle axonopathy (Figure 1D), sclerosis of the tunica media and adventitia in arterial vessels (Figure 1A,B,E). The proportion of muscle tissue in histological sections was statistically higher in GMFCS II-III and GMFCS V groups (pairwise comparisons: GMFCS II-III and GMFCS V (*p* = 0.79), GMFCS IV and GMFCS V (*p* = 0.04), GMFCS II-III and GMFCS IV (*p* = 0.02)). Connective tissue predominance was revealed in GMFCS IV group (pairwise comparisons: GMFCS II-III and GMFCS V (*p* = 0.68), GMFCS IV and GMFCS V (*p* = 0.01), GMFCS II-III and GMFCS IV (*p* = 0.03)). The percentage of adipose tissue was comparable across all study groups (*p* = 0.81).

No significant intergroup differences in sarcomere length were recorded (*p* = 0.14). However, all groups demonstrated Z-line distances (telophragma) in myofibrils noticeably below the 2.64 μm—the minimal threshold required for maximal muscle force generation (Figure 1F): GMFCS II-III—1.9 (1.8; 2.1) μm, GMFCS IV—2.1 (1.9; 2.4) μm, and GMFCS V—1.9 (1.7; 2.0) μm.

Slow-twitch oxidative fibers predominated in the gracilis muscle of GMFCS IV patients (pairwise comparisons: GMFCS II-III and GMFCS V (*p* = 0.761), GMFCS IV and GMFCS V (*p* = 0.001), GMFCS II-III and GMFCS IV (*p* = 0.041). Conversely, fast-twitch fibers were more abundant in GMFCS II-III and GMFCS V groups (pairwise comparisons: GMFCS II-III and GMFCS V (*p* = 0.927), GMFCS IV and GMFCS V (*p* < 0.001), and GMFCS II-III and GMFCS IV (*p* < 0.001)). Morphometric characteristics of gracilis muscle are presented in Table 2.

A statistically significant reduction in creatine phosphokinase activity was observed in intraoperative gracilis muscle specimens from GMFCS V patients compared to both GMFCS II-III and GMFCS IV groups (pairwise comparisons: GMFCS II-III and GMFCS V (*p* = 0.004), GMFCS IV and GMFCS V (*p* = 0.048), and GMFCS II-III and GMFCS IV (*p* = 0.358)). Biochemical characteristics of gracilis muscle tissue are provided in Table 3.

## 4. Discussion

The role of spasticity in the development of muscle contractures remain undiscussable [2]. On the other hand, understanding the intrinsic features associated with the changes in morphology and metabolism of skeletal muscle in cerebral palsy could elucidate the underlying mechanisms responsible for muscle alterations in CP and help clinicians to apply variable treatments that take these aspects into account [1,4,6]. Furthermore, heterogeneity of the CP phenotype can stimulate development of personalized medicine through the understanding of muscle pathomorphology and function on an individual basis.

Spasticity, resulting from damage to central motor neurons in the cerebral cortex, is diagnosed in 85–91% of patients with cerebral palsy and represents the most common neurological syndrome [25]. The widespread use of intramuscular botulinum neurotoxin A injections in these children is aimed at reducing hypertonia, causing dose-dependent reversible chemodenervation [6]. Frequent application of this method has been associated with reduced metabolically active muscle tissue, increased fibrofatty infiltration in striated muscle tissue, and reduced force-generating capacity of contractile tissue, resulting in worsening motor impairments [6,7]. In such a context, strength training exercises incorporated into rehabilitation programs may further stimulate catabolic processes and cause dystrophic changes in myosymplasts, eventually leading to muscle fibrosis progression [4,6,7]. The observed increase in connective tissue proportion within the gracilis muscle of GMFCS IV children likely reflects the above-mentioned processes, though our study is underpowered to confirm these conclusions.

Current literature soundly documents the degenerative processes occurring in skeletal muscle following efferent innervation disorders [26]. Research has also demonstrated reduced force generation capacity and microscopic structural reorganization of contractile tissue under conditions of impaired blood supply or gas exchange [27,28]. The axonopathy and angiopathy observed in the gracilis muscle as part of the research may represent possible pathomorphological substrates of myopathy in cerebral palsy. In this context, skeletal muscle fibers spared from dystrophic degeneration assume compensatory motor functions, resulting in their overload and hypertrophy [29]. Myosymplast transformation may account for increased muscle tissue proportion observed histologically in GMFCS V patients, as reported in the literature but requiring further investigation [29]. The progressive fibrofatty replacement of striated muscle tissue in children with severe CP motor impairments can condition an unfavorable rehabilitation prognosis related to limited motor function recovery, as well as low responsiveness to physiotherapy or other intervention approaches [30].

As per the sliding filament theory, muscle contraction occurs within sarcomeres due to the sliding of actin filaments relative to myosin filaments, powered by ATP energy [7]. Maximum force generation occurs within a narrow range of myofilament overlap, corresponding to basal contractile unit lengths between 2.64–2.81 μm [6,7]. The Z-line distances in myofibrils across all patient groups were found to be below the lower limit of normal. We hypothesize, this may indirectly indicate sarcomere shortening and weakness due to myocyte degeneration, an increased proportion of fibro-adipose tissue in the endomysium/perimysium, and abnormalities in connectin [6,7,31]. However, the measurements were performed in vitro on chemically fixed fragments of contractile tissue following release from passive tension, which causes shortening of the contractile units. This limitation affects the reliability of results and highlights the need for further research [7].

Children with cerebral palsy frequently demonstrate impaired oromotor function, increasing their risk of malnutrition and nutrition deficiency-related illnesses [32]. The primary manifestations of compromised trophic status involve delayed physical and cognitive development, metabolic dysfunction, osteoporosis, and secondary sarcopenia [4,33]. Severe nutritional deficiencies predominantly affect patients with profound motor impairments [24], a finding supported by our research results demonstrating lower body weight and Quetelet index in GMFCS V children compared to other groups.

A sedentary lifestyle, marked muscle hypertonia, and limb immobilization due to orthopedic interventions contribute to reduced excitation transmission in neuromuscular synapses; reduced intensity of cellular metabolic processes; and, consequently, altered ratios of fast (glycolytic) and slow (oxidative) fibers in striated muscle tissue [28]. As a result, approximately 75% of patients with cerebral palsy lose their ambulatory skills or ability to use assistive devices with age, leading to a significant decline in their daily mobility and autonomy [4]. In our study, children classified as GMFCS V group mostly exhibited strong, fast-twitch, anaerobic, creatine phosphokinase (alactic), and glycolytic (lactic) fibers, indicating rapid fatigue in these patients and limited capacity for prolonged strength exercises. This finding is supported by the observed low activity of creatine phosphokinase in the contractile tissue, which catalyzes creatine phosphate formation. The deficiency of creatine phosphate limits rapid ATP regeneration during substrate-level phosphorylation and all energy-demanding processes in the myosymplast [34]. The highest proportion of slow-twitch aerobic muscle fibers in GMFCS IV group demonstrates their favorable rehabilitation potential given the predominance of oxidative phosphorylation in myocyte mitochondria as the most efficient process for adenosine triphosphate synthesis. On the other hand, less significant morphological and biochemical changes revealed in muscles of children with level II and III of GMFCS can be interpreted as expecting a favorable response to physical rehabilitation and, in particular, strengthening exercises.

Thus, muscular morphological and biochemical abnormalities, alongside nutritional deficiencies and primary upper motor neurons dysfunction, reduce the ability of children with cerebral palsy to perform strength exercises as part of rehabilitation programs. The current study aimed to identify and correlate structural and biochemical muscle changes within the hip adductors (gracilis muscle) to disease severity in children undergoing soft tissue releases as a part of multilevel surgery. Understanding the pathophysiological mechanisms underlying the development of spastic muscle helps justify the most appropriate planning of therapeutic physical activity and develop novel interventions for the management of muscle contractures in these patients.

### Study Limitations

Several limitations of this study must be recognized. The primary constraint is the limited sample size within each comparison group. Although this enabled the identification of potential factors limiting the functional activity of children with different levels of motor impairment, it prevented extrapolation of the findings to the broader population. An additional limitation is the focus on a single muscle, which is another factor restricting the extrapolation of the results. Given that sarcomere length depends on the muscle fiber, passive tension, and joint position at the time of intraoperative sampling, and due to the lack of in vivo Z-line distance measurements, we were unable to establish the baseline length of the contractile units or assess the children’s rehabilitation potential. A further study should take into account other multiple interacting factors beyond muscle morphology, including neural control, motor planning, cognition, motivation, comorbidities, and the intensity and specificity of intervention.

## 5. Conclusions

The structural and metabolic abnormalities observed in gracilis muscle of patients with spastic cerebral palsy indicates profound functional muscular dysfunction, representing one of the factors limiting children’s motor ability. The morphological and biochemical alterations in the striated muscle of CP children correlate with severity of motor dysfunction conditioned by the primary upper motor neuron disorders. Less significant changes in muscles in ambulatory children reflect favorable basis for physical therapy.

## Figures and Tables

**Table 1:** Characteristics of groups, Me (Q1; Q3).

Group	** Sex		Weight (kg)	Weight/Age Percentile	Z-Score	* Quetelet Index (kg/m^2^)	BMI/Age Percentile	Z-Score	** EDACS (Level)		
	Male	Female							I	II	III
GMFCS II-III (n = 8)	5 (62.5)	3 (37.5)	32 (17; 37)	75 (59; 75)	0.6 (0.2; 0.7)	15.7 (1.9)	33 (25; 43)	−0.4 (−0.7; −0.2)	7 7 (87.5)	1 1 (12.5)	0 0 (0)
GMFCS IV (n = 8)	7 (87.5)	1 (12.5)	31 (25; 38)	75 (65; 86)	0.6 (0.4; 1.1)	16.9 (3.3)	50 (19; 50)	0 (−0.9; 0)	5 5 (62.5)	2 2 (25)	1 1 (12.5)
GMFCS V (n = 8)	5 (62.5)	3 (37.5)	17 (15; 20)	31 (25; 56)	−0.5 (−0.7; 0.2)	13.4 (1.4)	25 (20; 33)	−0.7 (−0.9; −0.4)	3 3 (37.5)	3 3 (37.5)	2 2 (25)
*p*	0.45		0.01	0.11	0.11	0.02	0.39	0.37	0.33		
η^2^	n/a		0.38	n/a	n/a	0.34	n/a	n/a	n/a		

* Mean and standard deviation (SD); ** number of patients, n (%).

**Table 2:** Morphometric parameters of gracilis muscle by group, Me (Q1; Q3).

Group	Muscle Fiber Diameter (μm)	Percentage Composition of Morphological Components in Gracilis Muscle (%)						Sarcomere Length (μm)	Fiber Ratio	
		Muscular Tissue	Connective Tissue	Adipose Tissue	Perimysial Vessels	Perimysial Nerves	Neuromuscular Spindles		Type I	Type II
GMFCS II-III (n = 8)	27 (21; 35)	61 (58; 69)	30 (25; 36)	3 (2; 8)	0.3 (0.2; 0.6)	0.01 (0; 0.04)	0 (0; 0.1)	1.9 (1.8; 2.1)	33 (25; 44)	66 (56; 75)
GMFCS IV (n = 8)	30 (23; 39)	41 (37; 52)	47 (45; 57)	1 (0.3; 13)	0.8 (0.6; 1)	0	0	2.1 (1.9; 2.4)	38 (28; 50)	62 (50; 71)
GMFCS V (n = 8)	31 (23; 51)	67 (46; 70)	29 (24; 36)	0.6 (0.3; 11)	0.6 (0.3; 0.8)	0	0	1.9 (1.7; 2.0)	33 (23; 44)	67 (57; 77)
*p*	0.67	0.03	0.03	0.81	0.18	0.36	0.41	0.14	<0.001	<0.001
η^2^	n/a	0.3	0.31	n/a	n/a	n/a	n/a	n/a	0.63	0.64

**Table 3:** Biochemical characteristics of gracilis muscle by group, Me (Q1; Q3).

Parameter	GMFCS II-III	GMFCS IV	GMFCS V	*p*	η^2^
Myosin, mg/g tissue	* 64.7 (9.4)	* 60.3 (8.5)	* 64.7 (7.2)	0.492	n/a
Proteolytic activity, U/mg protein	3.3 (2.6; 6.9)	4.5 (3.4; 7.7)	4.3 (3.2; 5.8)	0.717	n/a
LDH, U/mg protein	3647 (2470–6240)	3998 (2056–5006)	2197 (1402–2706)	0.278	n/a
CPK, U/mg protein	89.676 (77.165–104.130)	78.468 (55.150–96.402)	43,786 (34.347–60.318)	0.012	0.38
Glucose, mmol/g tissue	1.2 (0.9–2.2)	1.0 (0.5–1.4)	1.4 (1.1–2.3)	0.362	n/a
Lactate, mmol/g tissue	12.8 (7.4–17.8)	10.0 (8.2–12.3)	11.1 (9.0–12.2)	0.778	n/a

* Mean and standard deviation (SD).

## Data Availability

The data presented in this study are available on request from the corresponding author. The data are not publicly available due to privacy and ethical restrictions.

## Abbreviations

The following abbreviations are used in this manuscript:

ANOVA	One-way analysis of variance
ATP	Adenosine triphosphate
BMI	Body Mass Index
CP	Cerebral Palsy
CPK	Creatine phosphokinase
EDACS	Eating and Drinking Ability Classification System
GMFCS	Gross Motor Function Classification System
LDH	Lactate dehydrogenase
M (SD)	Mean Standard Deviation
Me	Median
n/a	Not applicable
PTAH	Phosphotungstic Acid Hematoxylin
Q1 & Q3	First and third quartiles
Z-scores	Standard deviation scores
